# Earnings and Financial Compensation from Social Security Systems Correlate Strongly with Disability for Multiple Sclerosis Patients

**DOI:** 10.1371/journal.pone.0145435

**Published:** 2015-12-22

**Authors:** Andrius Kavaliunas, Michael Wiberg, Petter Tinghög, Anna Glaser, Hanna Gyllensten, Kristina Alexanderson, Jan Hillert

**Affiliations:** 1 Department of Clinical Neuroscience, Karolinska Institutet, Stockholm, Sweden; 2 Department of analysis and prognosis, Swedish Social Insurance Agency, Stockholm, Sweden; 3 Red Cross University College, Stockholm, Sweden; University of Oxford, UNITED KINGDOM

## Abstract

**Background:**

Multiple sclerosis (MS) patients earn lower incomes and receive higher benefits. However, there is limited knowledge of how this is correlated with their disability.

**Objective:**

To elucidate sources and levels of income among MS patients with different disability, assessed with the Expanded Disability Status Scale.

**Methods:**

A total of 7929 MS patients aged 21–64 years and living in Sweden in 2010 were identified for this cross-sectional study. Descriptive statistics, logistic and truncated linear regression models were used to estimate differences between MS patients regarding earnings, disability pension, sickness absence, disability allowance, unemployment compensation, and social assistance.

**Results:**

The average level of earnings was ten times lower and the average level of health- related benefits was four times higher when comparing MS patients with severe and mild disability. MS patients with severe disability had on average SEK 166,931 less annual income from earnings and SEK 54,534 more income from benefits compared to those with mild disability. The combined average income for MS patients was 35% lower when comparing patients in the same groups. The adjusted risk ratio for having earnings among MS patients with severe disability compared to the patients with mild disability was 0.33 (95% CI 0.29–0.39), while the risk ratio for having benefits was 1.93 (95% CI 1.90–1.94).

**Conclusions:**

Disease progression affects the financial situation of MS patients considerably. Correlations between higher disability and patient income were observed, suggesting that earnings and benefits could be used as measures of MS progression and proxies of disability.

## Introduction

Multiple sclerosis (MS) is one of the most common causes of neurological disability in young adults, having a significant socioeconomic impact for patients, which is further magnified because of the typical early age of MS onset [1–3].

Within the MS population, the spectrum of disability ranges from essentially unaffected to highly disabled. The most common measure utilised to assess physical disability is the Expanded Disability Status Scale (EDSS) [4], which is based on a standardized neurological examination, however, is non-linear and subjective in nature.

A literature review on the economic burden of MS concluded that there was a significant increase in costs associated with an increase in disease severity as measured by EDSS [5]. In a recent study [6], we reported that in 2010, both MS patients and matched controls from the total population in Sweden received most of their income through earnings followed by income from disability pension and sickness absence. However, MS patients had 15% lower earnings and 33% higher benefits than the controls. A main limitation of the previous study was the lack of clinical information, such as disease development and disability. To include such information would make it possible to describe the impact of MS on different types of income, as well as to enhance the knowledge on the possibility of using income as a proxy for MS disability.

Accordingly, the aim of this study was to examine sources and levels of income among MS patients with different disability level, as well as to identify other factors influencing income.

## Materials and Methods

A cross-sectional population based study was conducted, linking data from the following two sources:

1. The nationwide *Swedish Multiple Sclerosis Register* (SMSreg) was used to obtain information about individuals diagnosed with MS, age at MS onset, and data from the clinical visits including the dates and EDSS scores. SMSreg runs on government funding only and is used in all Swedish neurology departments. It is designed to assure quality health care for MS patients and has been active since 2001. Currently, SMSreg includes data on 14,500 of Sweden’s estimated 17,500 prevalent patients with MS [7, 8].
2. The *Longitudinal Integration Database for Health Insurance and Labour market Studies* (LISA) held by Statistics Sweden was used to obtain information on socio-demographic variables (age, sex, living region in the country, family composition, type of living area, country of birth, education) and annual amounts of six sources of incomes: earnings, disability pension, sickness absence, disability allowance, unemployment compensation, and social assistance in Swedish Crowns (SEK) for individuals aged 21–64 and living in Sweden during 2010 [9].

The unique personal identification number assigned to all residents in Sweden was used to conduct the linkage.

In this study, the following inclusion criteria regarding MS patients were used:

- At least one clinical visit with EDSS score recorded in 2010. If a patient had more than one clinical visit that year, the median EDSS value was calculated. If a patient did not have a clinical visit in 2010, the mean EDSS value was imputed from the last value in the two years before (2008 and 2009) and the first value in the two years after (2011 and 2012). MS patients who had no EDSS score in 5 years (2008–2012) were excluded.
- Age: 21–64 years in 2010.
- No missing values in the variables used for the analysis.

Sources of incomes were defined as follows:

- Earnings. Income from work including salaries, self-employment, and sick pay from the employer (in most cases day 2–14 of a sick-leave spell) [6, 9].
- Disability pension. Replacement for up to 64% of lost income due to permanent or long-term reduced work capacity due to disease or injury at 25%, 50%, 75% and 100% of ordinary work hours, ages 19–64 years [6, 9].
- Sickness absence. Replacement for up to 80% of lost income due to temporary reduced work capacity due to disease or injury at 25%, 50%, 75%, or 100% of ordinary work hours, ages 16–64 years [6, 9].
- Disability allowance. Compensation for extra expenditures in daily living due to permanent disability, ages 19–64 years [6, 9].
- Unemployment compensation. Replacement for up to 80% of lost income due to unemployment. Requires 12 months of employment before unemployment [6, 9].
- Social assistance. Municipal means-tested cash benefits to low-income households to ensure a minimum level of living standard [6, 9].

To evaluate the effect of disability on the levels of income, patients were stratified into four groups: those with mild (EDSS 0–3.5), moderate mild (EDSS 4–5.5), moderate severe (EDSS 6–6.5), and severe (EDSS 7–9.5) disability.

### Statistical analyses

Descriptive statistics were used to describe, in absolute and relative terms, levels of income from the six different sources. One-way analysis of variance (ANOVA) was used to compare continuous variables (income data) between the groups stratified by EDSS. For the categorical variables, a Chi-square test was used. Spearman’s rank correlation coefficient (r_s_) was estimated to observe correlation between 1) having or not having income and EDSS; 2) income as continuous variables and EDSS.

The truncated linear regression models with left truncation for having no earnings and no benefits were used to estimate the differences between MS patients with different disability regarding their income for those who had it. Differences in level of income were calculated for patients who had earnings (>0) and benefits (>0) in 2010 and expressed as percentage for the disability levels. Univariate and multivariate logistic regression analyses were performed to estimate the probability for having earnings and benefits. The estimates were interpreted as odds ratios (OR) with 95% confidence intervals (CI). OR from logistic regression were converted into crude estimates of the adjusted risk ratios [10, 11]. Differences were defined as statistically significant for p values lower than 0.05. Nagelkerke R-square was used to assess how regression models predict variability.

The project was approved by the Regional Ethical Review Board of Stockholm. Given informed consent is mandatory to enter patients’ clinical data to SMSreg. All patient records/information was anonymized and de-identified prior to analysis.

## Results

In total, 8350 MS patients aged 21–64 years and living in Sweden in 2010, were identified. Of those, 421 patients with any missing data (mainly age at MS onset) were excluded from further analysis. Descriptive data of 7929 MS patients, stratified by disability level can be found in S1 Table. The mean age of the patients was 45.0 ± 10.9 years and mean age at onset of the disease was 32.2 ± 9.8 years. The majority (77.9%) of the patients were above 36 years, 72.7% were women, 90.0% were born in Sweden, 89.8% had at least secondary education, and 75.5% lived in larger cities or medium-sized municipalities. When stratified by disability level, 67.5% of the patients had mild, 11.5% had moderate mild, 10.7% had moderate severe, and 10.3% had severe disability (S1 Fig). The average EDSS score was 3.2 ± 2.4 with median 2.5. Patients with more severe disease were older (p < 0.001, one-way ANOVA). As expected, the mean age was higher with disease progression. With greater disability, the proportion of men was also higher, e.g., 26.5% in the mild disability group and 34.2% in the severe disability group. Secondary education was the most common educational level among MS patients, overall (46.8%) and in the different disability groups, while the percentage of persons with lower education increased from 8.2% to 18.6% and the percentage of persons with higher education decreased from 46.5% to 31.6% with greater disability. The most common family composition was to live with a partner and with children (38.3% overall), but the percentage of this category decreased with greater disability from 44.1% to 17.3% and the most common family composition among the most disabled patients was to be single and without children (52.7%). Geographical region, type of living area, and country of birth did not vary substantially between different EDSS groups.

The majority of patients received income in form of earnings (70.7%) and benefits (64.9%) (S1 Table). The average level of earnings was ten times lower (SEK 214,090 and SEK 21,870) and the average level of health-related benefits was four times higher (SEK 34,950 and SEK 141,980) when comparing patients with severe (EDSS 7–9.5) and mild (EDSS 0–3.5) disability. The mean income from disability pension and disability allowance was higher among those with greater disability.

**Fig 1:**
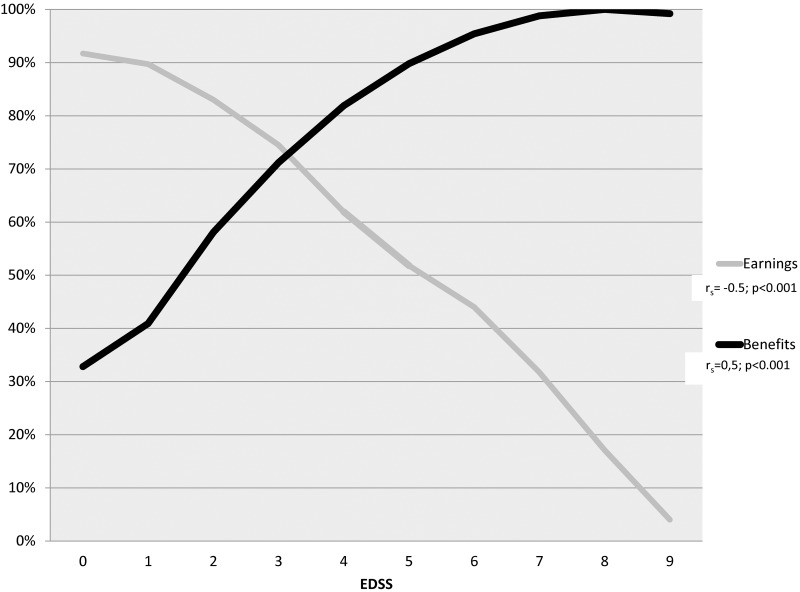
Percentages of MS patients who had earnings and got benefits, by different disability levels.

The proportion of MS patients who had earnings differed by disability level and gradually decreased from 91.7% at EDSS 0 to only 4.0% at EDSS 9 (r_s_ = -0.5; p<0.001), while the opposite trend was observed for receiving benefits—from 32.8% at EDSS 0 to 99.2% at EDSS 9 (r_s_ = 0.5; p<0.001) (Fig 1). Already at EDSS 4, more patients had income from benefits than from earnings.

**Fig 2:**
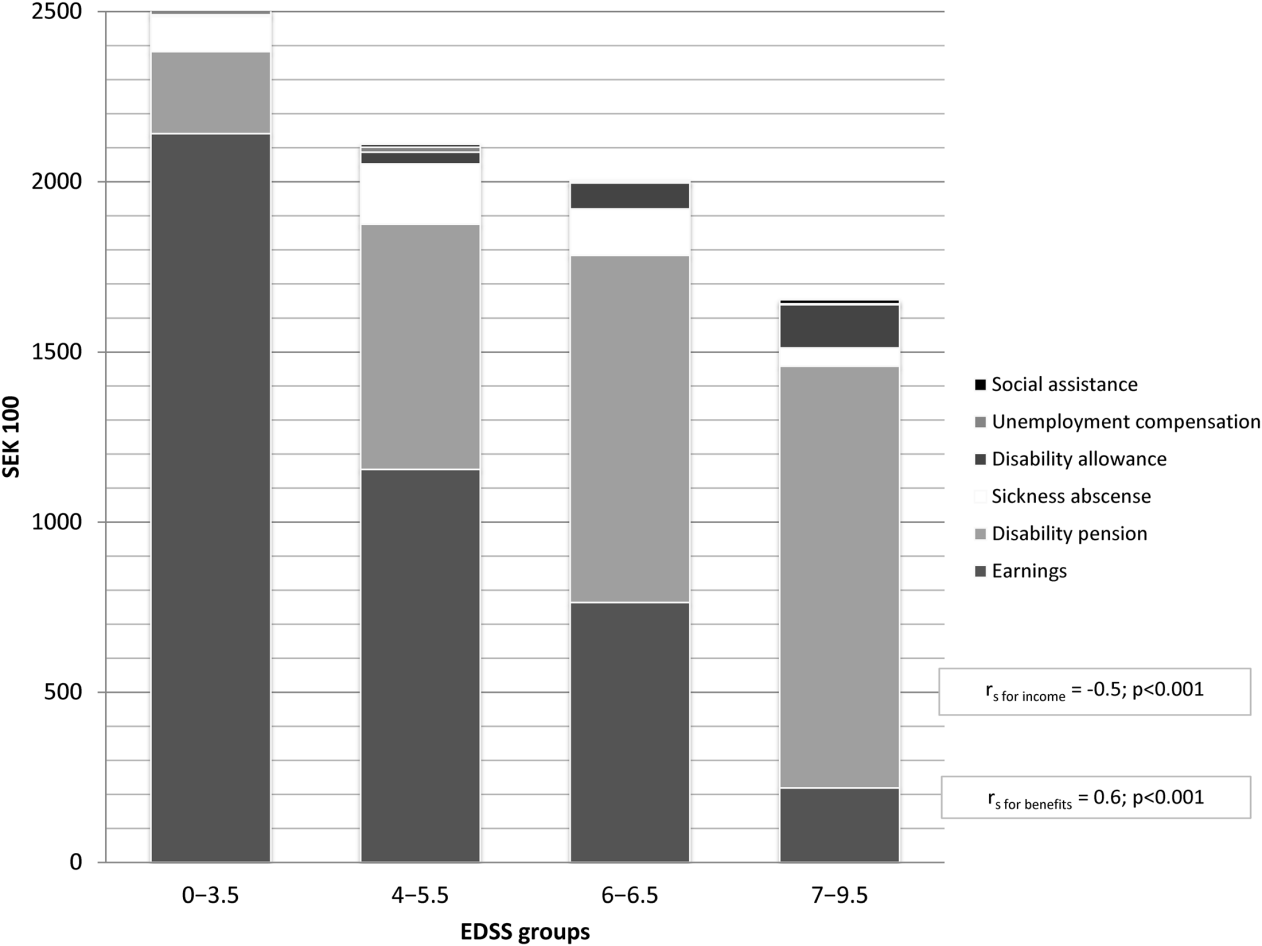
Average annual income by their different types for MS patients with different disability in 2010.

The combined average income in 2010 for MS patients decreased with greater disability from SEK 252,644 for the mild disability group to SEK 165,373 for the severe disability group (r_s_ = -0.5; p<0.001) (Fig 2). Also, earnings contributed to 85% of the income for the mild disability group and this proportion was only 13% for the severe disability group, where the main type of income was disability pension that contributed to 75% of all income.

Table 1 shows the results of the multivariate logistic regression analysis presented as ORs for having income for MS patients with different disability (full results, S2 Table). The estimated ORs for having earnings among MS patients with severe disability compared to the patients with mild disability was 0.07 (95% CI 0.06–0.09), while the estimated OR for having benefits was 89.13 (95% CI 36.73–216.28) when comparing the same groups. Age, age at onset, sex, education, family composition and type of living area were significant factors influencing the odds for having income, while geographical region was not. Country of birth was an important factor that significantly decreased odds to have income for the MS patients who were born outside Sweden, but it did not influence odds to receive benefits. As our outcomes were rather common (e.g., more than half of the patients in the mild disability group had earnings and received benefits), to avoid the possible misleading interpretation of OR, we converted these estimates into approximate risk ratios, for example, adjusted risk ratio for having earnings among MS patients with severe disability compared to the patients with mild disability was 0.33 (95% CI 0.29–0.39), while the risk ratio for having benefits was 1.93 (95% CI 1.90–1.94).

**Table 1:** Odds ratios with approximated risk ratios for having income among MS patients with different disability levels.

	Earnings (>0)		Benefits (>0)	
Disability level	OR (95% CI)	Adjusted risk ratio (95% CI)	OR (95% CI)	Adjusted risk ratio (95% CI)
EDSS **0–3.5**	1	1	1	1
EDSS **4–5.5**	0.32 (0.27–0.37)	0.75 (0.71–0.79)	4.06 (3.33–4.96)	1.58 (1.52–1.64)
EDSS **6–6.5**	0.21 (0.17–0.24)	0.63(0.57–0.67)	12.72 (9.09–17.80)	1.81 (1.76–1.85)
EDSS **7–9.5**	0.07 (0.06–0.09)	0.33(0.29–0.39)	89.13 (36.73–216.28)	1.93 (1.90–1.94)
Nagelkerke R-square	0.382		0.367	

ORs adjusted for age, age at MS onset, sex, geographical region, family composition, type of living area, country of birth, education.

Estimates of magnitudes of the incomes showed that MS patients with severe disability had 58.8% lower earnings and 92% higher benefits than the MS patients with mild disability (Table 2). As mentioned, a number of factors beside the level of disability affected their odds to have income for MS patients. In order to adjust for these, as well as to take into account zero values, that are abundant in these types of data, we conducted a linear regression analysis with left truncation for zero values (Table 3). This showed that MS patients with severe disability had on average SEK 166,931 less annual income from earnings and SEK 54,534 more income from benefits compared to those with mild disability (p <0.001) who had earnings and received benefits. A similarly significant effect on income was seen for other factors. Overall, female sex had the largest negative impact, besides disability, on earnings (SEK -88,866 on average compared to men) (full results, S3 Table).

**Table 2:** The differences in level of income among MS patients by disability level.

	Earnings >0			Benefits >0		
Disability level	n	Mean in SEK 100	Percentage change	n	Mean in SEK 100	Percentage change
EDSS **0–3.5** (mild)	4531	2526.4	Reference	2741	752.1	Reference
EDSS **4–5.5** (moderate mild)	530	1993.1	-21.1%	778	1122.9	+49.3%
EDSS **6–6.5** (moderate severe)	373	1735.9	-31.3%	809	1300.9	+73.0%
EDSS **7–9.5** (severe)	172	1041.6	-58.8%	814	1443.8	+92.0%
Total/Average	5606	2377.8	-5.9%	5142	1004.1	+33.5%

**Table 3:** Truncated linear regression for level of income in 2010 among MS patients with different disability level.

Disability level	Earnings >0			Benefits >0		
	Coefficient	SE	95% CI	Coefficient	SE	95% CI
EDSS **0–3.5**	Reference			Reference		
EDSS **4–5.5**	-658.11	80.64	-816.21− -500.02	285.50	20.70	244.92–326.08
EDSS **6–6.5**	-945.04	96.24	-1133.71− -756.36	422.74	21.14	381.30–464.18
EDSS **7–9.5**	-1669.31	138.03	-1939.91–1398.72	545.34	22.16	501.90–588.78

Adjusted for age, age-squared, age at MS onset, sex, geographical region, family composition, type of living area, country of birth, education

## Discussion

In this cross-sectional study, based on clinical and register data, we assessed income of MS patients in relation to disability level and other factors, and found significant correlations between greater disability and lower earnings and higher income from benefits.

Several studies on the socioeconomic situation of MS patients have shown that they have lower levels of income compared to those without MS [1, 6, 12]. In a register-based study from Denmark, the authors found that the employment rate for the MS patients steadily decreased within time after they received a diagnosis of MS with a corresponding increase in social transfers, whereas old-age retirement was similar in both groups [1]. Also, employed patients earned only two thirds of the income of employed control subjects.

Since research on MS patients’ financial situation still is very limited, our study contributes with knowledge on different types of income in relation to disability level. Descriptive data of our study population, regarding socioeconomic variables and EDSS scores, were well in line with the few previous similar studies [6, 13, 14]. In a recent study we found that 61.7% of the MS patients were on disability pension in 2005 [14], which is similar to the results of the present study regarding 2010 (64.9% of the patients received some type of benefits). Here we also show that the proportion of patients receiving some type of benefits was twice as high in the group with severe disability—where almost everyone received benefits—compared to the group of patients with mild disability. Such factors as female sex, older age, lower educational level, not born in Sweden, or living in smaller municipalities were shown to be associated with a higher OR for disability pension [14, 15]. Here we found that the same factors influenced income for MS patients with the most advanced disease. Their OR for receiving some type of benefit was as high as 89.13 (95% CI 36.73–216.28) while their OR for income through earnings was 0.07 (95% CI 0.06–0.09) when compared to the patients with mild disability. Although the approximate risk ratios showed a lower, but still significant, effect size, 1.93 (95% CI 0.29–0.39) and 0.33 (1.90–1.94) respectively, that provides a more interpretable perception of increased and decreased relative risk.

The multivariate logistic regression analysis showed Nagelkerke R-square value of 0.382 for earnings and 0.367 for benefits, whereas running these models without EDSS would give values of 0.24 and 0.25, respectively, proving that EDSS is an important component to assess in analyzes of factors influencing income of MS patients. In terms of numbers, this means that MS patients with severe disability on average had SEK 166,931 less annual income from earnings and SEK 54,534 more income from benefits (≈EUR 17,600 and EUR 5,700 respectively, using the average exchange rate of EUR 1 = SEK 9.5 in 2010 [16]) compared to those with mild disability. One of the limitations in this type of study was the abundance of zero values that prevented from analysis of data with a multivariate linear regression, thus we used truncated linear regression to calculate estimates for patients who had earnings (>0) and received benefits (>0), but it is important to note that at severe disability only 21% had income from earnings.

Although the highest total average income of the MS patients was attributable to earnings, followed by disability pension and sickness absence, which was previously reported,[6] we clearly see that this situation differed with level of disability: patients with mild and moderate mild disability (EDSS 0–5.5) mostly had earnings, whereas patients with moderate severe and severe disability (EDSS 6–9.5) had their main source of income from disability pension. To the previously reported findings [6], that MS patients had considerably higher levels of average disability allowance, while the matched controls from the general population had higher levels of unemployment compensation, we can now add that these types of benefits correlated with higher disability level: average disability allowance was 24 times higher among MS patients with severe disability when compared to the patients with mild disability (SEK 126,800 and SEK 5,300, respectively); unemployment compensation was 10 times lower when comparing the same groups (SEK 29,500 and SEK 3,100, respectively). The combined average income for MS patients was 35% lower in the severe disability group compared with the mild disability group.

Compared to most countries, Sweden has a universal health insurance system with broad coverage and high benefit levels, nevertheless, chronic diseases cause adverse social and economic consequences [17]. The reduced work capacity for individuals diagnosed with MS has in previous studies been associated with higher risk for labour market marginalization and higher usage of social security systems than in the general population [1, 12]. Other studies dealing with the economic burden of MS have shown the socio-economic challenge for people living with MS and the health systems [13, 18–20], which is also true for this study.

A study on employment in MS using logistic regression to determine predictors of work loss, showed that educational attainment and age emerged as the only demographic characteristics significantly predictive of employment loss, with greater levels of education being protective against work loss and older age predicting work loss, but gender not being a significant factor [21]. Interestingly, EDSS was one of the few independent predictors for the costs of MS that remained significant when analysed using a multivariate regression model [22].

In this cross-sectional study we looked at different sources of income as well as their levels in relation to MS disability and were able to include important clinical information from the SMSreg. To exploit more of the available clinical data, we also included EDSS values from the two years before (2008 and 2009) and two years after (2011 and 2012). The proportion of imputed EDSS values from 2008–2009 and 2011–2012 was quite substantial, 49.1%, however, we do not think it had an impact on the results, as MS usually has a long-term progression, for example, median time for conversion to a secondary progressive course is about 19 years and the median time to reach irreversible disability levels of DSS 4, 6, and 7 are 8, 20, and 30 years respectively [23]. We also used stratification of EDSS into 4 groups that makes the analysis less sensitive to changes between particular scores during the years. Moreover, if a patient did not have a clinical visit during 2010, it would be reasonable to assume that the patient’s disability did not change.

The strengths of our study include a large sample and the population-based register approach where two databases were linked enabling use of sociodemographic and clinical data of high quality and allowing stratifications by disability level.

Despite the many covariates analysed and included into the multivariate logistic model, including EDSS, they explained about 40% of the variance, suggesting that other factors influence income among MS patients. It is tempting to speculate that disabilities insufficiently covered by EDSS, such as disabling fatigue and cognitive symptoms, may play important roles. If so, one could argue that socioeconomic parameters such as income, earnings and benefits, may indeed give a more comprehensive account of the consequences of MS for the individual. There are, however, other factors that may influence the association between disability related to MS and the income/benefit ratio, such as concomitant diseases and factors related to behaviour, attitudes, and access to care, which warrants further studies. We suggest that these parameters should be explored further as potentially novel outcomes in a variety of research questions in MS including studies of prognostic factors and the evaluation of the impact of medications.

## Conclusions

MS progression significantly affects the financial situation of MS patients in Sweden. Individuals with severe disability had 59% lower earnings and 92% higher benefits than patients with mild disability. In spite of the compensatory benefit in Sweden, the overall income difference was 35% between those groups. The high correlation between EDSS and both earnings and benefits indicate that these could be used as proxies for disability in registry studies investigating factors of importance for MS progression.

## Supporting Information

S1 Fig Distribution of MS patients by disability level. Stratification of the subjects into four severity groups is indicated by the dashed lines.
(DOCX)

S1 Table Descriptive data of MS patients with different disability levels, aged 21–64 who lived in Sweden in 2010. (DOCX)

S2 Table Univariate and multivariate logistic regression for having income among MS patients with different disability levels. (DOCX)

S3 Table Truncated linear regression for level of income in 2010 among MS patients with different disability level. (DOCX)
