# Supplementary material for: Earnings and Financial Compensation from Social Security Systems Correlate Strongly with Disability for Multiple Sclerosis Patients
Source: PLoS One. 2015 Dec 22;10(12):e0145435. doi: 10.1371/journal.pone.0145435 (PMC4691204; doi:10.1371/journal.pone.0145435)
Supplement: S1 Fig — Stratification of the subjects into four severity groups is indicated by the dashed lines. (DOCX) [file pone.0145435.s001.docx]

**S1 Fig. Distribution of MS patients by disability level. Stratification of the subjects into four severity groups is indicated by the dashed lines**
