# Supplementary material for: Earnings and Financial Compensation from Social Security Systems Correlate Strongly with Disability for Multiple Sclerosis Patients
Source: PLoS One. 2015 Dec 22;10(12):e0145435. doi: 10.1371/journal.pone.0145435 (PMC4691204; doi:10.1371/journal.pone.0145435)
Supplement: S1 Table — (DOCX) [file pone.0145435.s002.docx]

**S1 Table. Descriptive data of MS patients with different disability levels, aged 21−64 who lived in Sweden in 2010**

|  | **All** selected  (N=7929) | **Disability by EDSS groups** | | | |
| --- | --- | --- | --- | --- | --- |
|  |  | **Mild**  **(0−3.5)**  (n=5347) | **Moderate mild (4−5.5)** (n=915) | **Moderate severe (6−6.5)** (n=848) | **Severe**  **(7−9.5)**  (n=819) |
| **Gender ***  Male  Female | 2243 (28.3%)  5686 (71.7%) | 1415 (26.5%)  3932 (73.5%) | 283 (30.9%)  632 (69.1%) | 265 (31.3%)  583 (65.8%) | 280 (34.2%)  539 (65.8%) |
| **Age** (mean, ±SD) * | 45.0 ± 10.9 | 42.0 ± 10.5 | 49.0 ± 9.6 | 52.3 ± 8.9 | 52.7 ± 8.4 |
| **Age groups ***  21-24  25-34  35-44  45-54  55-64 | 237 (3.0%)  1307 (16.5%)  2226 (28.1%)  2331 (29.4%)  1828 (23.1%) | 226 (4.2%)  1184 (22.1%)  1742 (32.6%)  1458 (27.3%)  737 (13.8%) | 10 (1.1%)  68 (7.4%)  221 (24.2%)  321 (35.1%)  295 (32.2) | 0 (0.0%)  32 (3.8%)  148 (17.5%)  264 (31.1%)  404 (47.6%) | 1 (0.1%)  23 (2.8%)  115 (14.0%)  288 (35.2%)  3921 (47.9%) |
| **Age at MS onset** (mean, ±SD) * | 32.2 ± 9.8 | 32.1 ± 9.6 | 33.1 ± 10.5 | 33.9 ± 10.4 | 30.2 ± 9.6 |
| **Geographical region ***  East  South/West  North | 3341 (42.1%)  3011 (38.0%)  1577 (19.9%) | 2312 (43.2%)  1978 (37.0%)  1057 (19.8%) | 390 (42.6%)  342 (37.4%)  183 (20.0%) | 303 (35.7%)  371 (43.8%)  174 (20.5%) | 336 (41.0%)  320 (39.1%)  163 (19.9%) |
| **Family composition ***  With partner, no children  With partner, with children  Single, no children  Single, with children | 1403 (17.7%)  3036 (38.3%)  2826 (35.6%)  664 (8.4%) | 726 (13.6%)  2357 (44.1%)  1796 (33.6%)  468 (8.8%) | 224 (24.5%)  310 (33.9%)  297 (32.5%)  84 (9.2%) | 256 (30.2)  227 (26.8%)  301 (35.5%)  64 (7.5%) | 197 (24.1%)  142 (17.3%)  432 (52.7%)  48 (5.9%) |
| **Type of living area ***  Larger cities  Medium-sized municipalities  Smaller municipalities | 3428 (43.2%)  2559 (32.3%)  1942 (24.5%) | 2324 (43.5%)  1751 (32.7%)  1272 (23.8%) | 410 (44.8%)  257 (33.7%)  248 (27.1%) | 332 (39.2%)  286 (33.7%)  230 (27.1%) | 362 (44.2%)  265 (32.4%)  192 (23.4%) |
| **Country of birth**  Sweden  Other Nordic  Other EU-25  Other | 7138 (90.0%)  193 (2.4%)  155 (2.0%)  443 (5.6%) | 4819 (90.1%)  117 (2.2%)  100 (1.9%)  311 (5.8%) | 826 (90.3%)  23 (2.5%)  19 (2.1%)  47 (5.1%) | 763 (90.0%)  26 (3.1%)  18 (2.1%)  41 (4.8%) | 730 (89.1%)  27 (3.3%)  18 (2.2%)  44 (5.4%) |
| **Education ***  Lower  Secondary  Higher | 872 (11.0%)  3713 (46.8%)  3344 (42.2%) | 438 (8.2%)  2423 (45.3%)  2486 (46.5%) | 133 (14.5%)  464 (50.7%)  318 (34.8%) | 149 (17.6%)  418 (49.3%)  281 (33.1%) | 152 (18.6%)  408 (49.8%)  259 (31.6%) |
| **Earnings >0 *** | 5606 (70.7%) | 4531 (84.7%) | 530 (57.9%) | 373 (44.0%) | 172 (21.0%) |
| **Benefits >0 *** | 5142 (64.9%) | 2741 (51.3%) | 778 (85.0%) | 809 (95.4%) | 814 (99.4%) |
| **Earnings** (mean in SEK 100) * | 1681.2 | 2140.9 | 1154.5 | 763.6 | 218.7 |
| **Health related benefits ***  (mean in 100 SEK) | 621.7 | 349.5 | 932.1 | 1232.2 | 1419.8 |
| Disability pension * | 482.9 | 241.3 | 720.3 | 1019.6 | 1239.4 |
| Sickness absence * | 110.0 | 102.9 | 177.2 | 136.3 | 53.7 |
| Disability allowance * | 28.8 | 5.3 | 34.7 | 76.3 | 126.8 |
| **Benefits related to low income *** (mean in SEK 100) | 29.4 | 36.0 | 22.7 | 8.9 | 15.2 |
| Unemployment compensation * | 22.3 | 29.5 | 14.6 | 4.2 | 3.1 |
| Social assistance | 7.1 | 6.6 | 8.1 | 4.7 | 12.1 |

* p < 0.05, compared patients in different disability groups; ANOVA or Chi-square tests.
