# Supplementary material for: Earnings and Financial Compensation from Social Security Systems Correlate Strongly with Disability for Multiple Sclerosis Patients
Source: PLoS One. 2015 Dec 22;10(12):e0145435. doi: 10.1371/journal.pone.0145435 (PMC4691204; doi:10.1371/journal.pone.0145435)
Supplement: S2 Table — (DOCX) [file pone.0145435.s003.docx]

**S2 Table. Univariate and multivariate logistic regression for having income among MS patients with different disability levels**

|  | **Odds (earnings >0)** | | | | **Odds (benefits >0)** | | | |
| --- | --- | --- | --- | --- | --- | --- | --- | --- |
|  | Univariate | | Multivariate | | Univariate | | Multivariate | |
|  | OR | 95% CI | OR | 95% CI | OR | 95% CI | OR | 95% CI |
| EDSS **0−3.5** | 1 |  | 1 |  | 1 |  | 1 |  |
| EDSS **4−5.5** | 0.25 | 0.21−0.29 | 0.32 | 0.27−0.37 | 5.40 | 4.47−6.53 | 4.06 | 3.33−4.96 |
| EDSS **6−6.5** | 0.14 | 0.12−0.17 | 0.21 | 0.17−0.24 | 19.72 | 14.24−27.32 | 12.72 | 9.09−17.80 |
| EDSS **7−9.5** | 0.05 | 0.04−0.06 | 0.07 | 0.06−0.09 | 154.78 | 64.15−373.48 | 89.13 | 36.73−216.28 |
| **Age** | 0.94 | 0.94−0.95 | 0.96 | 0.95−0.97 | 1.07 | 1.06−1.07 | 1.06 | 1.05−1.07 |
| **Age at MS onset** | 1.00 | 0.99−1.00 | 1.02 | 1.01−1.02 | 1.01 | 1.01−1.02 | 0.97 | 0.97−0.98 |
| **Sex:**  Male  Female | 1  0.88 | 0.79−0.98 | 1  0.63 | 0.55−0.72 | 1  1.46 | 1.32−1.62 | 1  2.06 | 1.82−2.33 |
| **Geographical region:**  East  South/West  North | 1  1.02  1.01 | 0.92−1.14  0.88−1.15 |  |  | 1  1.12  1.36 | 1.01−1.24  1.19−1.54 | 1  1.07  1.15 | 0.95−1.21  0.97−1.35 |
| **Family composition:**  With partner, no children  With partner, with children  Single, no children  Single, with children | 1  3.18  1.25  1.59 | 2.76−3.66  1.09−1.42  1.31−1.94 | 1  1.50  0.74  0.91 | 1.26−1.79  0.62−0.88  0.72−1.15 | 1  0.37  0.53  0.77 | 0.32−0.43  0.46−0.61  0.63−0.96 | 1  0.94  1.17  1.48 | 0.79−1.12  0.96−1.41  1.16−1.88 |
| **Type of living area:**  Larger cities  Medium-sized municipalities  Smaller municipalities | 1  0.94  0.81 | 0.84−1.05  0.72−0.91 | 1  0.90  0.80 | 0.78−1.03  0.69−0.92 | 1  1.21  1.66 | 1.09−1.35  1.47−1.87 | 1  1.17  1.42 | 1.02−1.33  1.21−1.66 |
| **Country of birth:**  Sweden  Other Nordic  Other EU-25  Other | 1  0.47  0.59  0.49 | 0.35−0.62  0.43−0.82  0.40−0.59 | 1  0.54  0.44  0.26 | 0.38−0.77  0.30−0.66  0.20−0.33 | 1  1.22  1.06  1.13 | 0.89−1.66  0.76−1.48  0.92−1.38 |  |  |
| **Education:**  Lower  Secondary  Higher | 1  2.40  5.41 | 2.07−2.79  4.61−6.35 | 1  1.99  4.55 | 1.67−2.38  3.77−5.50 | 1  0.48  0.23 | 0.39−0.58  0.19−0.28 | 1  0.57  0.28 | 0.46−0.70  0.23−0.35 |
| Nagelkerke R-square |  | | 0.382 | |  | | 0.367 | |
