# Supplementary material for: Earnings and Financial Compensation from Social Security Systems Correlate Strongly with Disability for Multiple Sclerosis Patients
Source: PLoS One. 2015 Dec 22;10(12):e0145435. doi: 10.1371/journal.pone.0145435 (PMC4691204; doi:10.1371/journal.pone.0145435)
Supplement: S3 Table — (DOCX) [file pone.0145435.s004.docx]

**S3 Table. Truncated linear regression for level of income in 2010 among MS patients with different disability level**

|  | **Earnings >0** | | | **Benefits >0** | | |
| --- | --- | --- | --- | --- | --- | --- |
|  | Coefficient | SE | 95% CI | Coefficient | SE | 95% CI |
| EDSS **0-3,5** | Reference | | | Reference | | |
| EDSS **4-5,5** | -658.11 | 80.64 | -816.21− -500.02 | 285.50 | 20.70 | 244.92−326.08 |
| EDSS **6-6,5** | -945.04 | 96.24 | -1133.71− -756.36 | 422.74 | 21.14 | 381.30−464.18 |
| EDSS **7-9,5** | -1669.31 | 138.03 | -1939.91−1398.72 | 545.34 | 22.16 | 501.90−588.78 |
| **Age** | 192.61 | 18.67 | 156.01− -229.22 | 35.50 | 5.82 | 24.09−46.91 |
| **Age squared** | -1.99 | 0.22 | -2.42− -1.57 | -0.24 | 0.06 | -0.37− -0.12 |
| **Age at MS onset** | 11.73 | 3.03 | 5.80−17.66 | -0.23 | 0.84 | -1.87− -1.41 |
| **Sex:**  Male  Female | Reference  -888.66 | 51.32 | -989.28− -788.05 | Reference  -101.00 | 16.26 | -132.88− -69.13 |
| **Geographical region:**  East  South/West  North | Reference  -337.26  -242.09 | 51.54  70.15 | -438.30− -236.21  -379.61− -104.58 | Reference  -15.94  -26.78 | 15.75  20.54 | -46.81−14.93  -67.04−13.48 |
| **Family composition:**  With partner, no children  With partner, with children  Single, no children  Single, with children | Reference  54.95  -35.59  -88.79 | 78.26  79.91  105.65 | -98.47−208.37  -192.25−121.07  -295.90−118.32 | Reference  -42.85  31.79  28.75 | 21.94  20.51  29.09 | -85.86−0.15  -8.41−71.99  -28.27−85.78 |
| **Type of living area:**  Larger cities  Medium-sized municipalities  Smaller municipalities | Reference  -376.13  -539.89 | 56.29  66.73 | -486.47− -265.78  -670.70− -409.08 | Reference  -7.98  4.95 | 17.38  19.59 | -42.06−26.10  -4.95−43.36 |
| **Country of birth:**  Sweden  Other Nordic  Other EU-25  Other | Reference  -101.29  -63.28  -441.75 | 168.22  178.47  113.02 | -431.07−228.49  -413.15−286.58  -663.31− -220.20 | Reference  -11.38  -177.85  -56.51 | 44.00  49.95  30.52 | -97.62−74.87  -275.77− -79.94  -116.34−3.32 |
| **Education:**  Lower  Secondary  Higher | Reference  286.72  1021.97 | 93.32  93.54 | 103.77−469.67  838.60−1205.35 | Reference  -23.37  -72.38 | 21.07  22.32 | -64.68−17.93  -116.14− -28.62 |
